# Editorial: Prognostic research and precision oncology in upper tract urothelial carcinoma-volume II

**DOI:** 10.3389/fonc.2023.1276718

**Published:** 2023-09-05

**Authors:** Tsung-Mu Yang, Nirmish Singla, Chung-Hsin Chen, Jer-Tsong Hsieh, Hsin-Chih Yeh

**Affiliations:** ^1^ Department of General Medicine, Kaohsiung Medical University Hospital, Kaohsiung, Taiwan; ^2^ Brady Urological Institute, School of Medicine, Johns Hopkins Medicine, Baltimore, MD, United States; ^3^ Department of Urology, National Taiwan University Hospital, Taipei, Taiwan; ^4^ Department of Urology, University of Texas Southwestern Medical Center, Dallas, TX, United States; ^5^ Department of Urology, Kaohsiung Municipal Ta-Tung Hospital, Kaohsiung, Taiwan; ^6^ Department of Urology, Kaohsiung Medical University Hospital, Kaohsiung, Taiwan; ^7^ Department of Urology, School of Medicine, College of Medicine, Kaohsiung Medical University, Kaohsiung, Taiwan; ^8^ Graduate Institute of Medicine, College of Medicine, Kaohsiung Medical University, Kaohsiung, Taiwan

**Keywords:** upper tract urothelial carcinoma, precision oncology, surgical wait time, nomogram, immunochemotherapy, CCAAT/enhancer-binding protein delta

Upper tract urothelial carcinoma (UTUC) is an aggressive but rare urological cancer. Prognostic models in UTUC have largely been derived from clinical and pathological factors, although more recently, investigators have turned to the molecular landscape to gain prognostic information. Compared to UTUC, bladder urothelial carcinoma (UC) is more common. As UTUC shares many genomic alterations with bladder UC (1), studies of bladder UC have provided important reference for the management of UTUC. These studies have helped facilitate personalized management strategies. In this Research Topic, four articles, including two original articles, one case report, and one review article, explore the prognostic factors in UC with an emphasis towards advancing precision medicine.

The gold standard treatment of localized UTUC is radical nephroureterectomy (RNU) with bladder cuff resection. However, there can be various reasons for delayed surgery (2). The outbreak of coronavirus disease-19 (COVID-19) has significantly impacted medical practices globally, resulting in prolonged surgical wait times (SWT). In a multicenter retrospective cohort involving 1,251 Asian (mainly Taiwanese) patients, Wu et al. find that a SWT of more than 90 days is independently associated with worse overall survival (OS) and disease-free survival. This study is the first large-scale study of its kind in Asia, and the authors’ important findings emphasize the negative impact of prolonged SWT on survival.

In a study on >3,000 patients with high-grade bladder UC, Li et al. establish a prognostic nomogram by leveraging the SEER database (3). This nomogram includes six variables: T stage, positive lymph nodes, age, chemotherapy, regional lymph nodes examined, and primary tumor size. Both internal and external validation demonstrate the excellent discrimination and calibration ability of the nomogram. This nomogram may assist urologists in optimizing follow-up plans, determining subsequent treatment options, and improving outcomes in this group of patients.

Xu et al. demonstrate the application of precision medicine in the systemic treatment of UTUC. Platinum-based chemotherapy is considered the first-line therapy for locally advanced and metastatic UC, while immune checkpoint inhibitors (ICIs) are recommended for patients who are ineligible for cisplatin or have refractory disease after first-line chemotherapy. In order to optimize first-line treatment regimens, recent trials have focused on combining ICIs with chemotherapy, known as immunochemotherapy (ICT). However, the results of these trials were inconsistent (4–6). In their case report, Xu et al. present a case of highly aggressive UTUC. Through pathologic and molecular analyses, their patient’s tumor is classified as luminal-infiltrated subtype, characterized by TP53/MDM2 and ERBB2 mutations, non-mesenchymal state, and immune-infiltrated contexture, suggesting potential responsiveness to ICIs. Their patient receives ICT with gemcitabine, carboplatin, and the off-label PD-1 inhibitor sintilimab, followed by sintilimab maintenance monotherapy, leading to a sustained complete response over 2 years. This case highlights ICT as a promising option for first-line therapy in selected patients.

The final article explores the genetic predisposition of UC. CCAAT/enhancer-binding protein delta (CEBPD), a transcription factor, functions as a potent regulator for cell differentiation, proliferation, motility, growth arrest, and cell death. Notably, CEBPD overexpression is associated with a poor prognosis in UC, which has been found to have oncogenic functions in multiple regulation pathways. The review article by Chan et al. provides a comprehensive reappraisal of the regulatory mechanisms of CEBPD in UC progression and its characteristics in response to cisplatin treatment. CEBPD drives cancerization by influencing glycolytic metabolism shift and angiogenesis through multiple mechanisms involving MYC, hsa-miR-429, HK2, VEGFA, and MMP2. However, the role of upregulated CEBPD after cisplatin treatment remains uncertain. Two studies have proposed the potential association of CEBPD with cisplatin resistance (7, 8), while another study suggested its possible role in anti-tumorigenesis (9). Further investigations are needed to clarify this controversy. Lastly, their review sheds light on the involvement of CEBPD in the tumor microenvironment, underscoring the necessity for additional exploration in this field.

This Research Topic provides readers with novel insights into the prognostic factors of UTUC to help guide current clinical practice and serve as an essential juncture for future research.

## Author contributions

TY: Writing – review & editing, Writing – original draft. NS: Writing – review & editing, Validation. CC: Validation, Writing – review & editing. JH: Validation, Writing – review & editing. HY: Validation, Writing – review & editing, Supervision.
